# A Non-redundant Function of MNS5: A Class I α-1, 2 Mannosidase, in the Regulation of Endoplasmic Reticulum-Associated Degradation of Misfolded Glycoproteins

**DOI:** 10.3389/fpls.2022.873688

**Published:** 2022-04-19

**Authors:** Xiaoxia Sun, Chenchen Guo, Khawar Ali, Qian Zheng, Qiang Wei, Yumeng Zhu, Li Wang, Guishuang Li, Wenjuan Li, Bowen Zheng, Qunwei Bai, Guang Wu

**Affiliations:** College of Life Sciences, Shaanxi Normal University, Xi’an, China

**Keywords:** *SBI3*, ERAD, *BRI1*, MNS4, MNS5

## Abstract

Endoplasmic Reticulum-Associated Degradation (ERAD) is one of the major processes in maintaining protein homeostasis. Class I α-mannosidases MNS4 and MNS5 are involved in the degradation of misfolded variants of the heavily glycosylated proteins, playing an important role for glycan-dependent ERAD *in planta*. MNS4 and MNS5 reportedly have functional redundancy, meaning that only the loss of both MNS4 and MNS5 shows phenotypes. However, MNS4 is a membrane-associated protein while MNS5 is a soluble protein, and both can localize to the endoplasmic reticulum (ER). Furthermore, MNS4 and MNS5 differentially demannosylate the glycoprotein substrates. Importantly, we found that their gene expression patterns are complemented rather than overlapped. This raises the question of whether they indeed work redundantly, warranting a further investigation. Here, we conducted an exhaustive genetic screen for a suppressor of the *bri1-5*, a brassinosteroid (BR) receptor mutant with its receptor downregulated by ERAD, and isolated *sbi3*, a suppressor of *bri1-5* mutant named after *sbi1* (suppressor of *bri1*). After genetic mapping together with whole-genome re-sequencing, we identified a point mutation G343E in AT1G27520 (MNS5) in *sbi3*. Genetic complementation experiments confirmed that *sbi3* was a loss-of-function allele of *MNS5*. In addition, *sbi3* suppressed the dwarf phenotype of *bri1-235* in the proteasome-independent ERAD pathway and *bri1-9* in the proteasome-dependent ERAD pathway. Importantly, *sbi3* could only affect BRI1/bri1 with kinase activities such that it restored BR-sensitivities of *bri1-5*, *bri1-9*, and *bri1-235* but not null *bri1*. Furthermore, *sbi3* was less tolerant to tunicamycin and salt than the wild-type plants. Thus, our study uncovers a non-redundant function of MNS5 in the regulation of ERAD as well as plant growth and ER stress response, highlighting a need of the traditional forward genetic approach to complement the T-DNA or CRISPR-Cas9 systems on gene functional study.

## Introduction

In eukaryotic cells, the endoplasmic reticulum (ER) is an important organelle for newly synthesized polypeptides. Since protein folding is not completely precise and easily affected by factors such as amino acid mutations, alterations in post-transcriptional and translational modifications, or biotic and abiotic stress, the newly proteins translocated into the ER are heterogeneous (Howell, 2013; Balchin et al., 2016). As such, a vast majority of the proteins are subjected to a sophisticated and flexible endoplasmic reticulum quality control (ERQC) system that detects the proteins and ensures their correct folding, posttranslational modifications, assembly, and secretion, otherwise the proteins will be degraded with the help of regulatory mechanisms such as molecular chaperones, sugar-binding lectins, and folding enzymes (Ellgaard and Helenius, 2003; Gidalevitz et al., 2013). As such, only terminally correctly folded proteins can reach their final destination (Ellgaard and Helenius, 2003; Strasser, 2018).

The majority of newly synthesized secretory and membrane proteins are N-glycosylated (Aebi, 2013). A specific sequence Asn-X-Ser/Thr (where X can be any amino acid except proline) is recognized by enzyme oligosaccharyltransferase complex (OST) (Kelleher and Gilmore, 2006), which integrates a three-branched tetradecasaccharide precursor Glc_3_Man_9_GlcNAC_2_ (glucose, mannose, and N-acetylglucosamine) from a dolichylpyrophosphate (Dol-PP) carrier to the selected asparagine residues on the nascent peptides (Helenius and Aebi, 2004; Pattison and Amtmann, 2009; Mohorko et al., 2011). Thus, the structure of N-linked glycan plays an important role in protein folding and quality control.

After the rapid removal of the two glucose residues by α-glucosidase I (GI) and α-glucosidases II (GII), the monoglucosylated N-glycan Glc1Man9GlcNAc2 will interact with the two ER-resident lectins, calnexin and calreticulin (CNX and CRT) (Trombetta, 2003; Helenius and Aebi, 2004; Deprez et al., 2005; Williams, 2006; D’Alessio et al., 2010; Stigliano et al., 2011; D’Alessio and Dahms, 2015). This slows the cleavage of the innermost glucose residue by GII, which liberates maturing Man9GlcNAc2-containing glycoproteins from CNX/CRT, thus terminating its folding process in the ER (Caramelo and Parodi, 2008). If the glycoproteins are not properly folded, they will be recognized and reglucosylated by the luminal enzyme UDP-glucose: glycoprotein glucosyltransferase (UGGT) and subjected to additional rounds of CNX/CRT cycle until the protein is fully mature (Sousa and Parodi, 1995; Parodi, 2000; Taylor et al., 2003; Helenius and Aebi, 2004; Caramelo and Parodi, 2007; Jin et al., 2007). The glycoproteins that fail terminally to acquire their native structure are retained in the ER and eventually are selected for a unique degradative mechanism known as ER-associated degradation (ERAD) (McCracken and Brodsky, 1996; Vembar and Brodsky, 2008; Christianson and Ye, 2014). Most of the previous ERAD studies were based on analysis obtained from yeast or mammals (Thibault and Ng, 2012; Christianson and Ye, 2014). The existence of similar ERAD mechanism has also been reported in plants (Strasser, 2018).

In Arabidopsis, the terminal α1,2 Man residue from C-branches of the misfolded glycoproteins is trimmed by the ER-localized α1,2 mannosidase 4 (MNS4) and α-mannosidase 5(MNS5) (Htm1 in yeast, EDEMs in mammals), generating Glc_0–1_Man_7–8_GlcNAc_2_ with a free α1,6 Mannose residue on the C-branch as an N-glycan ERAD signal (Lederkremer and Glickman, 2005; Quan et al., 2008; Clerc et al., 2009; Liebminger et al., 2009; Huttner et al., 2014b; Ninagawa et al., 2014; Schoberer et al., 2019). This glycan signal is recognized by EBS6/AtOS9 (YOS9 in yeast; OS-9 and XTP3-B in mammals) and Hrd3/Sel1L in plants (Denic et al., 2006; Gauss et al., 2006; Hirsch et al., 2009; Hosokawa et al., 2009; Yoshida and Tanaka, 2010; Liu et al., 2011; Su et al., 2011, 2012; Huttner et al., 2012; Ruggiano et al., 2014; Ohta and Takaiwa, 2015). Thus, terminally misfolded glycoproteins carrying an exposed α1,6-mannose residue may be recruited to a membrane-embedded Hrd1 complex (Carvalho et al., 2010; Baldridge and Rapoport, 2016), ubiquitination, and subsequent dislocation into the cytoplasm for degradation (Smith et al., 2011). The Hrd1 complex contains evolutionarily conserved components: EBS5/HRD3A (Liu et al., 2011; Su et al., 2011), Hrd1a/1b (Su et al., 2011), EBS6/AtOS9 (Huttner et al., 2012; Su et al., 2012), their associated E2 conjugase UBC32 (Cui et al., 2012), and plant-specific components: EBS7 (Liu et al., 2015) and PAWH1/PAWH2 (Lin et al., 2019).

In order to decipher the function of genes in a wide range of organisms, many different techniques have been developed over the years, but different approaches often give rise to different phenotypes. One paradoxical example is that the knockouts (via genetic inactivation) of a gene largely do not cause any obvious phenotypic symptoms, while the knockdowns (the reduction of expression) of the same gene exhibit severe biological defects. These phenomena have been previously observed in a number of model systems, including yeast (Jost and Weiner, 2015), Drosophila (Yamamoto et al., 2014), mouse (Dawlaty et al., 2011; Freudenberg et al., 2012), Zebrafish (Rossi et al., 2015; El-Brolosy et al., 2019; Ma et al., 2019), human cell lines (Lin et al., 2007, 2017; Hebbard et al., 2010; Wang et al., 2014; Speers et al., 2016), and Arabidopsis (Braun et al., 2008; Chen et al., 2014; Gao et al., 2015). These results suggest that genetic compensation in response to a gene knockout might occur (El-Brolosy and Stainier, 2017). A previous study had revealed that the ER-localized *MNS4* and *MNS5* accelerated the demannosylation of the C-branch to generate a terminal α1, 6-linked Man acting as the glycan signal for ERAD, and found that the null mutant *mns4-1* or *mns5-1* obtained by transfer DNA (T-DNA) insertion could not separately suppress the phenotypes of *bri1-5* and *bri1-9* mutants that have become excellent materials to study and understand the ERQC in plants (Jin et al., 2007; Hong et al., 2008, 2012). Yet, their double mutant led to the inhibition of the dwarfism of *bri1-5* and *bri1-9* (Huttner et al., 2014b). These results suggest that MNS4 and MNS5 are functionally redundant to each other by gene duplication or genetic compensation in response to a gene knockout, yet it has not been investigated.

Forward genetics is an effective molecular approach that has led to the identification of several important ERAD complex components in plants (Jin et al., 2007, 2009; Su et al., 2011, 2012; Hong et al., 2012; Liu et al., 2015). Here, we used the EMS-mutagenized approach to isolate another suppressor of *bri1-5* mutant (*sbi3*, suppressor *of bri1 3*) that carried a point mutation Gly343Glu in MNS5 (*AT1G27520*). We found that the dwarf phenotype of *bri1-5* and *bri1-9* was suppressed by the *sbi3* mutant. In addition, we found that *sbi3* inhibited the degradation of another recently reported ER-retained *BRI1* mutant, *bri1-235* (Hou et al., 2019), which has a single amino acid substitution from Ser to Phe at position 156 in the less conserved fourth LRR of BRI1 extracellular domain. Moreover, *sbi3* led to ER stress and was less tolerant to salt. Therefore, our finding demonstrates that *MNS5* has a non-redundant function in the regulation of plant growth, Interestingly, the reverse transcription PCR (RT-PCR) analysis reveals that the expression levels of *MNS4* and *MNS5* had no change in mutant *sbi3*, namely a lack of a genetic compensatory response in *sbi3*. Surprisingly, *mns5-1* produces no transcripts (Huttner et al., 2014b), suggesting that the genetic compensatory response in *mns5-1* might not be due to the upregulaton of both *MNS4* and *MNS5*. As a result, the cause of the non-redundant function in *MNS5* remains to be uncovered. Nevertheless, our finding provides a new avenue for further investigation of the ERAD *in planta* and raises awareness of the importance of using both forward and reverse genetic studies for gene functions *in planta*.

## Materials and Methods

### Isolation of *bri1-5* (Ws-2) and *bri1-5* Suppressor Mutants

The *bri1-5* (Ws-2) seeds were mutagenized with 0.4% ethyl methanesulfonate (Sigma Aldrich). The M2 seeds, derived from around 10,000 M1 plants, were screened on one-half-strength Murashige and Skoog medium. These seeds were stratified in the dark at 4°C for 4 days, and then grown in the light at 22°C for 1 day, in the dark at 22°C for 4 days, and in the light at 22°C for 1 day (Wu et al., 2011). After germination, the seedlings with long hypocotyls were transferred into the soil for continued growth under a 16 h-light/8 h-dark growth condition for 4 weeks in the greenhouse, and mature seeds were then collected. The potential suppressors were genotyped using a *bri1-5-*dCAPS marker to eliminate pollen or seed contamination. The derived *sbi3 bri1-5* homozygous mutants were back-crossed three times to eliminate any unlinked second-site mutations.

### Plant Materials and Growth Conditions

The *Arabidopsis thaliana* ecotypes Wassilewskija-2 (Ws-2) and Columbia-0 (Col-0) were used as the wild-type (WT) control in this study. The mutants *bri1-9*, *bri1-235*, *bri1-301*, *bri1-116*, *det2-1*, *cpd*, and *bin2-1* were used in the Col-0 background, the mutant *bri1-119* was in Enkheim-2 (En-2), and the mutant *bri1-5* was in Ws-2 background. The *sbi3* was discovered in the genetic screen for the extragenic suppressor of *bri1-5*. The mutant *sbi3 bri1-5* was crossed into *bri1-9*, *bri1-235*, *bri1-301*, *bri1-116*, *bri1-119*, *det2-1*, *cpd*, and *bin2-1* respectively, to obtain the different background double mutants for genetic analyses. The seeds were surfaced sterilized by washing for 5 min in 75% (v/v) ethanol containing 0.05% (v/v) Tween 20 and for 1 min in 5% NaClO, followed by three-five times washes with sterilized water. Under sterile conditions, the seeds were sown on ½ Murashige and Skoog (MS) medium and plated at 4°C for 2–3 days to break dormancy and increase uniform germination, the seeds were germinated in Petri dishes at 22°C with 70% humidity under long-day (16/8-h light/dark) photoperiod (∼120 μmol.m^–2.^s^–1^) condition. One week after germination, seedlings were transferred into soil and grown under the same controlled conditions.

### Pavement Cell Analysis: Microscopy and Image Analysis

To observe the profile of the pavement cells, 7-day-old cotyledons were stained in propidium iodide (PI, Sigma) (10 μg/mL in H_2_O) for 5–10 min and washed three times (10 min each) in deionized water. Stained cotyledons were fixed firmly in water on slides for microscopy. The pavement cells were imaged using a Leica TCS SP8 laser scanning confocal microscope. Images were obtained with × 40 objective for propidium iodide (PI) staining. Images were captured by the following setting: 1.0 μm z-step size, 561 nm laser excitation, and 590–630 nm emission. ImageJ software was used to measure the lobe length, neck width, perimeter, and area^1^. Circularity was analyzed as previously described by Zhang et al. (2011). At least 30 cells were used for the analysis. The data was recorded and the significance was analyzed using the Student’s paired *t*-test.

### Map-Based Cloning

Map-based cloning was performed as described previously (Lukowitz et al., 2000). The *sbi3 bri1-5* (Ws-2) grown in the greenhouse was crossed with Col-0, and the resulted F1 plants were germinated and allowed for self-pollination. The *bri1-5*–like seedlings with shorter hypocotyls were selected in the segregating F2 population, and grown in the greenhouse to obtain F3 seeds. To map the mutated locus, we screened for seedlings with long hypocotyls exhibiting the suppressed-*bri1-5* phenotype in the F3 generation. Genomic DNA from 50 to 100 individual seedlings of the mapping population was extracted. The mutation site was first mapped to a region close to the SSLP markers by Bulked Segregant Analysis using pooled DNA samples. simple sequence length polymorphisms (SSLP) markers were listed in Supplementary Table 1. Meanwhile, the Whole Genome Resequencing was also performed from the genomic DNA of the mapping population by Beijing Nuohe Zhiyuan Technology Co., Ltd. MutMap method was used to rapidly identify the suppressor gene (Abe et al., 2012), and a graph relating single-nucleotide protein (SNP) positions and SNP-index was generated for all 5 *Arabidopsis thaliana* chromosomes. The SNP index was defined as the ratio between the number of reads of a mutant SNP and the total number of reads corresponding to the SNP. The causative SNP should be shared by all the mutant plants, therefore, SNP-index = 1 harbored the gene responsible for the mutant phenotype, and 0.5 for the unlinked loci to the mutant phenotype.

### Generation of Constructs and Transgenic Plants

The *SBI3* and *sbi3* (G343E) were amplified by PCR from cDNA of WT and *sbi3*. Both amplified genes were subsequently cloned into a T-Vector PMD19 (TaKaRa), and the cloned genes were verified by DNA sequencing. Later, the T-vectors carrying the genes were digested with KpnI and BamHI and cloned into the binary vector pCHF3 that carries the 35S promoter and a synthetic gene for the green fluorescent protein (GFP) to obtain *p35S:SBI3-GFP* and *p35S:sbi3-GFP* constructs, respectively. These resulting constructs were first transformed individually into the *sbi3 bri1-5* mutants *via Agrobacterium tumefaciens* (GV3101)-mediated transformation using the floral-dipping method (Clough and Bent, 1998). The transformants were germinated and screened on 1/2 MS medium with kanamycin (50 mg/L). To confirm the transformants, the transgene from each transgenic line was sequence-verified.

### Transcript Analysis by RT-PCR

The two-week-old Arabidopsis seedlings were collected and ground in liquid nitrogen into a fine powder, and their total RNAs were extracted using the TRIzol reagent (Invitrogen) following the manufacturer’s instructions. First-strand complementary DNA (cDNA) was synthesized from 1 μg/2 mg of the total RNA using an M-MLV First Strand cDNA Synthesis Kit (Omega, TQ2501-02, Norcross, GA, United States). The cDNA was then amplified by a Semi-quantitative RT-PCR system with gene-specific primers for *CPD*, *DWF4*, *BAS1*, *BRI1*, and *ACTIN2* to study the expression levels. The PCR amplified cDNA fragments were separated by agarose gel electrophoresis, the *ACTIN2* was used as an internal control, RT-PCR experiment was repeated three times. All primers used for RT-PCR were given in Supplementary Table 1.

### Western Blot

Arabidopsis seedlings treated with or without kifunensine (Kif), cycloheximide (CHX), MG132 (Abcam), or 24-epibrassinolide (24-eBL, Sigma) were harvested and ground into fine powder in liquid nitrogen. The total protein was suspended in 2x SDS sample buffer [100 mM Tris, pH 6.8, 4% (w/v) SDS, 20% (v/v) glycerol, 0.2% (w/v) bromophenol blue, 2% (v/v) β-Mercaptoethanol] and denatured at 100°C for 10 min. The protein was then centrifuged for 5 min at the top speed, and the resulting supernatants were then resolved on 8% SDS-PAGE gel and transferred into nitrocellulose membrane (Pall Gelman). Anti-GFP antibody (1:1,000 dilution, TransGen, HT801) was used to detect GFP fusion proteins, Anti-BRI1 antibody (1:1,000 dilution, Agrisera) was used to detect the protein expression levels of BRI1/bri1, and BES1 antibody (1:3,000 dilution, kindly provided by J. Li, Lanzhou University, China) was used to detect the phosphorylation status of BES1.

### Endo H Treatment

Total proteins were extracted from the 2-week-old grown seedlings with 2×SDS sample buffer. The extracted proteins were denatured at 100°C for 10 min in 1×glycoprotein denaturing buffer and centrifuged for 10 min at 10,000 × *g*, the resulted supernatant was transferred into a new 1.5 mL tube and incubated with or without Endo H (NEB, New England Biolads) digestion in the 1 × G5 buffer for 1 h at 37°C following the manufacturer’s protocol (New England Biolabs). Both control and Endo H–treated samples were separated by SDS-PAGE and transferred into nitrocellulose membrane (Pall Gelman), and the proteins were quantified by Anti-BRI1 antibody.

## Results

### The *sbi3* Mutation Suppresses Dwarf Phenotypes of *bri1-5*, *bri1-235*, and *bri1-9*

**FIGURE 1:**
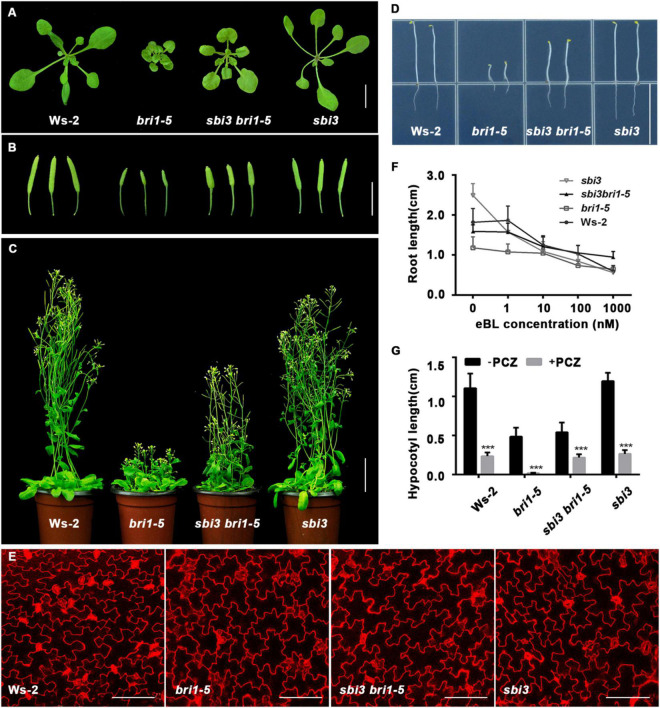
*sbi3* mutation partly suppresses *bri1-5* dwarfism. **(A)** Phenotypes of 2-week-old soil-grown seedlings of Ws-2, *bri1-5*, *sbi3 bri1-5*, and *sbi3*. Scale bar, 1 cm. **(B)** 2-month-old siliques of Ws-2, *bri1-5*, *sbi3 bri1-5*, and *sbi3*. Scale bar, 1 cm. **(C)** 2-month-old mature plants of Ws-2, *bri1-5*, *sbi3 bri1-5*, and *sbi3*. Scale bar, 3 cm. **(D)** Hypocotyl comparison of 5-day-old dark-grown seedlings of Ws-2, *bri1-5*, *sbi3 bri1-5*, and *sbi3*. Scale bar, 1.5 cm. **(E)** The morphology of cotyledon pavement cells of 7-day-old seedlings from Ws-2, *bri1-5*, *sbi3 bri1-5*, and *sbi3*. Cotyledons were stained by propidium iodide (PI). Scale bar, 100 μm. **(F)** Root growth phenotypes of 8-day-old Ws-2, *bri1-5*, *sbi3 bri1-5*, and *sbi3* seedlings grown in 1/2 MS medium with different 24-epibrassinolide (24-eBL) concentrations at 22°C under long-day (16/8-h light/dark) condition. Quantitative analysis of root length plotted as the line graph and displayed in panel **(F)**, *n* ≥ 30 seedlings. Error bar represents ± SD, three independent assays. **(G)** Hypocotyl growth phenotype of 5-day-old Ws-2, *bri1-5*, *sbi3 bri1-5*, and *sbi3* seedlings grown in 1/2 MS medium treated with or without 5 μM PCZ in the dark. Quantitative analysis of hypocotyl length of 5-day-old dark-grown seedlings plotted as histograms displayed in panel **(G)**, *n* ≥ 30 seedlings. ***P < 0.001 as two-way ANOVA with Sidak’s multiple comparisons test.

To identify the additional regulators of the ERAD, we performed a genetic screen for a sensitized extragenic suppressor in the *bri1-5* mutant that encodes a functionally competent receptor with a mutation in one of the cysteine pairs (C69Y) in the extracellular domain, serving a substrate of ERAD as a misfolded receptor kinase (Noguchi et al., 1999). We identified a putative EMS–mutagenized suppressor, *sbi3* that weakly suppressed the dwarf phenotype of *bri1-5* mutant. *sbi3 bri1-5* exhibited expanded rosette leaves with noticeable petioles at rosette stages (Figure 1A), long siliques, and long floral stems in the soil (Figures 1B,C), longer hypocotyls when grown in the dark (Figure 1D). In mutant *sbi3*, which was obtained by the hybridization of *sbi3 bri1-5* and Ws-2, the bolting time was 5–6 days earlier than that of the wild type Ws-2, and the number of rosette leaves was fewer in *sbi3* (Supplementary Figure 1). When the pavement cell shape in Ws-2, *bri1-5*, *sbi3 bri1-5*, and *sbi3* mutants was examined to understand the cellular mechanism underlying these phenotypes, we found that the mutant *bri1-5* exhibited a smaller area and perimeter of pavement cells and defective lobe structure. By contrast, the quantitative analysis results confirmed that pavement cell area and perimeter value were bigger, with smaller circularity value and narrower necks, in the double mutant *sbi3 bri1-5* than that of *bri1-5* (Figure 1E and Supplementary Figure 2). The morphological analysis of these phenotypes revealed that *sbi3* partially inhibited the dwarfing phenotypes of *bri1-5*. To determine whether BR responses were altered in the *sbi3 bri1-5* lines, we performed an exogenous BR (24-eBL) sensitivity assay on root growth to various concentrations of 0–1,000 nM. We found that the increasing concentrations of 24-eBL had little effect on the root elongation of the *bri1-5* seedlings, but greatly inhibited the root growth of the Ws-2, *sbi3*, and the *sbi3 bri1-5* seedlings in a dose-dependent manner (Figure 1F). Furthermore, we also examined the effect of exogenously applied Propiconazole (PCZ, Solarbio), a BR biosynthesis inhibitor that blocks the production of BRs, found that the hypocotyl growth of Ws-2, *sbi3 bri1-5*, and *sbi3* was more reduced by PCZ when compared to *bri1-5* (Figure 1G). These results confirmed that the sensitivity of double mutant *sbi3 bri1-5* to 24-eBL and PCZ had changed in comparison to *bri1-5* due to the point mutation in the *SBI3* gene.

**FIGURE 2:**
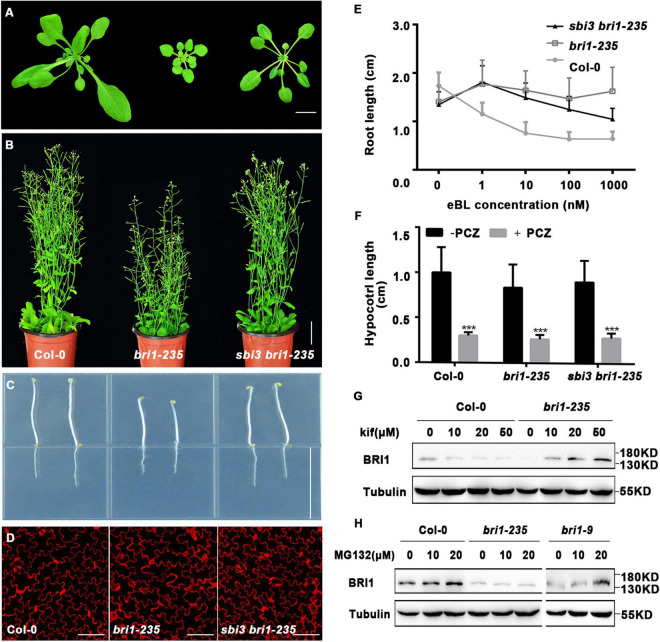
A mutant *sbi3* weakly suppresses *bri1-235* phenotypes. **(A)** Phenotypes of two-week-old soil-grown seedlings of Col-0, *bri1-235*, and *sbi3 bri1-235*. Scale bar, 1 cm. **(B)** Phenotypes of 2-month-old mature plants of Col-0, *bri1-235*, and *sbi3 bri1-235*. Scale bar, 3 cm. **(C)** Hypocotyl comparison of 5-day-old dark-grown seedlings of Col-0, *bri1-235*, and *sbi3 bri1-235*. Scale bar, 1.5 cm. **(D)** Morphology of cotyledon pavement cells of 7-day-old seedlings from Col-0, *bri1-235*, *sbi3 bri1-235*. Scale bar, 100 μm. (E) The 24-eBL-induced root inhibition assay. Quantitative measurements analysis of root length of 8-day-old Col-0, *bri1-235*, and *sbi3 bri1-235* seedlings were plotted as the line graph and displayed in panel **(E)**, *n* ≥ 30 seedlings. Error bar represents ± standard deviation (SD), three independent assays were performed with similar results. **(F)** Measurements of hypocotyl length of 5-day-old dark-grown in 1/2 MS medium treated with or without 5 μM PCZ seedlings were plotted as a histogram. ***P < 0.001. **(G)** Immunoblot analysis of BRI1 abundance in 2-week-old seedlings incubated with or without Kif for 24 h in liquid half-strength MS medium. The protein abundance of BRI1-235 was increased by treatment with Kif. **(H)** Immunoblot analysis of BRI1 proteins in 2-week-old seedlings of Col-0, *bri1-235*, and *bri1-9* supplemented with or without MG132 for 24 h in liquid half-strength MS medium.

Consistently, we found that the *sbi3 bri1-235* double mutant was also a larger and less compact rosette with taller stature at maturity compared to the *bri1-235* mutant (Figures 2A–D). As shown in Figure 2E, the treatment of *bri1–235* with increasing concentrations of brassinolide (24-eBL) had a less effect on root growth, compared to Col-0 and *sbi3 bri1–235* (Figure 2E). As expected, the hypocotyl of the Col-0, *bri1-235*, and *sbi3 bri1-235* seedlings exhibited hypersensitivity to PCZ (Figure 2F). We, therefore, speculated that *sbi3* mutant might broadly block the ERAD in the *bri1* mutants with ER-localization.

Given the fact that *bri1-235* is retained in the ER (Hou et al., 2019), one-week-old seedlings were transferred to a half-strength MS medium supplemented with or without 10 μM kifunensine (Kif), a widely used inhibitor of α1,2-mannosidases (Tokunaga et al., 2000), for continued growth for 9 days. We found that kif treatment exhibited less compact rosette, with short and radially swollen roots in *bri1-235* (Supplementary Figure 3A), which was consistent with findings on *bri1-5* and *bri1-9* mutants (Hong et al., 2008, 2009). The quantitative analysis of root length revealed similar responses to kif treatment for both *bri1-235* and wild-type seedlings (Supplementary Figure 3B).

The above findings suggest that the low BRI1-235 protein abundance in *bri1-235* seedlings could have been caused by ERAD. To test this hypothesis, we treated *bri1-235* and the wild-type seedlings with Kif. The result showed that Kif treatment significantly increased the abundance level of BRI1-235 in a dose-dependent manner, but had little effect on the BRI1 stability in Col-0 (Figure 2G), suggesting that ER-retained BRI1-235 undergoes ERAD, similar to the findings on *bri1-5* and *bri1-9* (Hong et al., 2008, 2009). It has been shown previously that ER-retained BRI1 mutant, *bri1-5*, was degraded by a proteasome-independent ERAD process (Hong et al., 2008), but ERAD of ectopically expressed BRI1-9:GFP involved proteasomes (Hong et al., 2009). To examine the ERAD mechanism of BRI1-235, we treated 2-week-old *bri1-235* seedlings with MG132, a widely used proteasome inhibitor that can prevent degradation of proteasome-dependent ERAD substrates (Schmitz and Herzog, 2004). The treatment analysis showed that the BRI1 protein abundance was increased drastically in MG132 treated Col-0 and *bri1-9* but not *bri1-235* (Figure 2H), indicating that BRI1 protein is degraded by a proteasome-independent ERAD process in *bri1-235*.

We then asked whether the *sbi3* could inhibit the proteasome-dependent ERAD in *bri1-9*. Indeed, we found that *sbi3* mildly rescued the dwarf phenotype of *bri1-9* mutant that had a small rosette, a short hypocotyl in the dark, small perimeter, and area, and short inflorescence stems of mature plants (Supplementary Figures 4A–D). In addition, the *sbi3 bri1-9* also showed increased sensitivity to exogenous BRs as compared to *bri1-9* (Supplementary Figure 4E).

### *sbi3* Is a New Mutant Allele of *MNS5*

To understand how *sbi3* mutation inhibited the dwarf phenotypes of *bri1-5*, *bri1-235* and *bri1-9*, we tried to clone the *SBI3* gene. We first backcrossed the *sbi3 bri1-5* double mutant to *bri1-5* to obtain the resulting F1 plants showing the *bri1-5* phenotype. The F2 plants showed an approximately 3:1 dwarf-to-normal phenotypic segregation, indicating that *sbi3* was a recessive mutant in a single gene. In order to isolate the *sbi3* gene, we employed the PCR-based positional cloning approach with pooled genomic DNA of 50-100 F3 that had *sbi3 bri1-5*-like seedlings derived from the mapping population and located the *SBI3* locus to a genomic region close to the SSLP marker chr1-9621kb on the top of chromosome 1 by Bulked segregant analysis (Supplementary Figure 5).

**FIGURE 3:**
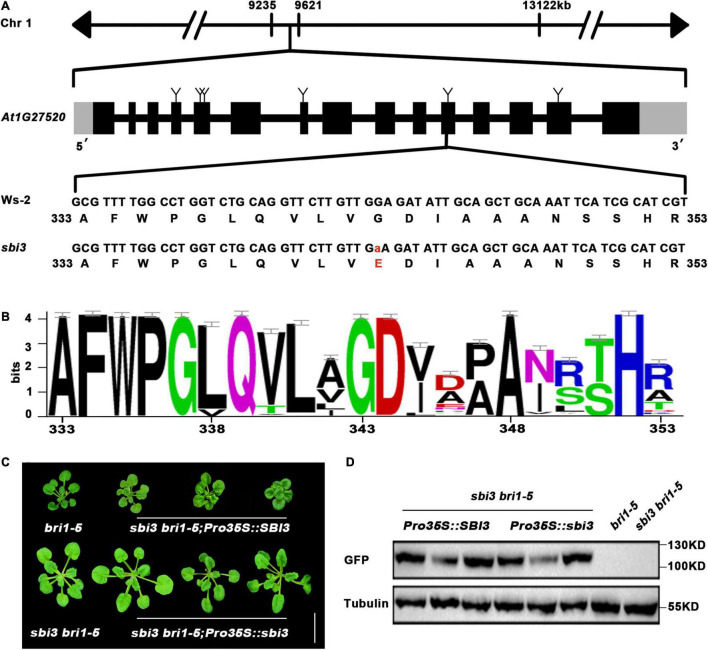
The molecular cloning of *sbi3.* **(A)** Schematic presentation of the mutation site in *sbi3*. **(B)** Sequence alignment of a small part of the SBI3 protein among different species. G residue at the 343rd position was highly conserved. **(C)** Three weeks-soil-grown plants of *bri1-5*, *sbi3 bri1-5*, three *SBI3*-complemented *sbi3 bri1-9* transgenic lines carrying an *SBI3* transgene driven by the 35S promoter, and three independent *sbi3* overexpression transgenic lines on *sbi3bri1-5* mutants served as control. Scale bar, 1 cm. **(D)** Protein expression levels of *bri1-5*, *sbi3 bri1-5*, and the corresponding transgenic plants with GFP tag shown in panel **(C)** were detected with anti-GFP antibody. Tubulin served as the loading control.

Subsequently, whole-genome re-sequencing (Supplementary Figure 6) revealed higher SNP-index values in the region between 8 and 10 Mb on chromosome 1. Two genes with SNP-index = 1 were identified in the candidate region by comparing with the published reference sequences of Col-0 and Ws-2. An SNP at position chr1-9,576,968 was located in a coding region of AT1G27570 (CDS: G499A, Protein: V167I). But according to the sequence alignment, it was not predicted to be the mutant position because it is not conservative. Another SNP (G343E) was at position chr1-9,560,890 bp, which was a single-nucleotide polymorphism substitution of G to A corresponded to the 11th exon in AT1G27520 (MNS5) between *sbi3 bri1-5* and *bri1-5*. *MNS5* encodes a glycosyl hydrolase family 47 known to be a critical ERAD component, suggesting that the non-synonymous mutation identified in G343A (for protein) accounts for suppressor *bri1-5 sbi3* phenotype. *SBI3/MNS5* encodes a polypeptide of 574-aa, which consists of 15 exons plus 14 introns (Figure 3A). Sequence alignment showed that this mutated G343 residue was absolutely conserved in SBI3/MNS5 among selected species shown in Supplementary Table 2 and Figure 3B.

That *MNS5* and *SBI3* are the same genes and were further confirmed by two additional experiments. First, this detected SNP was converted to a derived cleaved amplified polymorphic sequence (dCAPS) marker (Neff et al., 1998), which was used to confirm a tight genetic linkage between the G–A mutation and the *sbi3 bri1-5* phenotype in several F3 mapping populations. Second, we performed a genetic complementation experiment with *SBI3/MNS5*–GFP and *sbi3*-GFP constructs to rescue the *sbi3 bri1-5* mutant phenotypes, such that the phenotypes of three independent transgenic lines expressing *SBI3/MNS5*–GFP but not *sbi3*-GFP had similar phenotypes to *bri1-5*, confirming that *At1g27520 (MNS5)* was indeed *SBI3* (Figures 3C,D).

### The Molecular Mechanism of *sbi3*

**FIGURE 4:**
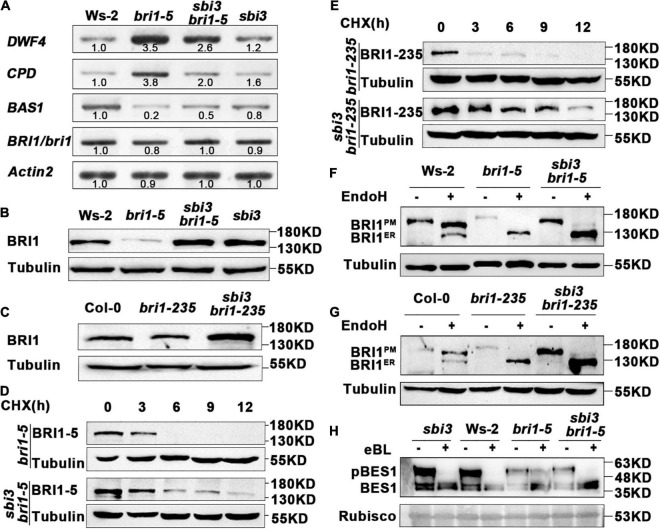
*sbi3* mutation inhibits the Endoplasmic Reticulum-Associated Degradation (ERAD) of *bri1-5* and *bri1-235* through a posttranscriptional mechanism. **(A)** The expression abundance of transcripts for BR receptor *BRI1* in Ws-2, *bri1-5*, *sbi3 bri1-5*, and *sbi3* seedlings was detected by semi-quantitative RT-PCR. The transcripts of *BRI1/bri1* in the wildtype (WT) or the mutant were similar. *Actin2* was used as an internal control. N = 3 biological replicates. **(B)** Western blot analysis of BRI1 protein abundance in Ws-2, *bri1-5*, *sbi3 bri1-5*, and *sbi3*. Extracts were prepared from 14-day-old seedlings grown in 1/2 MS medium. Specific antibodies: Anti-BRI1, Anti-Tubulin (control). **(C)** Immunoblot analysis of BRI1 protein abundance in Col-0, *bri1-235*, and *sbi3 bri1-235*. Specific antibodies: Anti-BRI1; Anti-Tubulin (control). **(D)** Immunoblot analysis of BRI-5 stability in *sbi3 bri1-5* with the anti-BRI1 antibody. Two-week-old seedlings were treated with 180 μM CHX for indicated incubation times. **(E)** Immunoblot analysis of BRI1-235 stability in *sbi3 bri1-235* with the anti-BRI1 antibody. Two-week-old seedlings were treated with 180 μM CHX for indicated incubation times. **(F)** Endoglycosidase H (EndoH) analysis of Ws-2, *bri1-5*, *sbi3 bri1-5*, *sbi3*. BRI1*^ER^* is the ER-localized proteins form, while BRI1*^PM^* denotes the localization of BRI1 proteins in the plasma membrane. **(G)** EndoH analysis of Col-0, *bri1-235*, and *sbi3 bri1-235*. **(H)** Immunoblotting of eBL induced dephosphorylation of *sbi3*, Ws-2, *bri1-5*, and *sbi3 bri1-5*. Rubisco served as a loading control.

To understand the underlying biochemical mechanism of which *sbi3* restores the BR receptor function of *bri1-5*, *bri1-235*, and *bri1-9*, we checked the expression level of these BR responsive genes (*DWF4*, *CPD*, and *BAS1*) in Ws-2, *bri1-5*, *sbi3 bir1-5*, and *sbi3* mutants by RT-PCR. It is known that *DWF4*, *CPD* (BR biosynthesis genes), and *BAS1* (BR inactivation genes) are sensitive feedback regulators for BR signaling (Tanaka et al., 2005). The results showed that the expression level of *DWF4* and *CPD* was significantly downregulated in wild-type, *sbi3*, and *sbi3 bri1-5* compared to the expression level of *DWF4* and *CPD* in *bri1-5* plants. On the contrary, the expression level of *BAS1* was upregulated in the wild-type, *sbi3* and *sbi3 bri1-5* compared to *bri1-5* (Figure 4A). However, the expression level of *BRI1/bri1-5* in *SBI3* and *sbi3* backgrounds was similar (Figure 4A). However, the immunoblot analysis revealed that the *sbi3* mutation greatly elevated the BRI1-5, BRI1-235, and BRI1-9 protein abundance level in double mutants (Figures 4B,C and Supplementary Figure 4F). Together, these data suggested that SBI3 could mediate the BRI1 abundance through a posttranscriptional mechanism.

To confirm that the increased BRI1-5 abundance was caused by increased synthesis or reduced degradation in *sbi3 bri1-5*, we treated 2-week-old seedlings of *bri1-5* and *sbi3 bri1-5* mutants with 180 uM CHX, a widely used protein biosynthesis inhibitor, and then analyzed the BRI1-5 abundance by immunoblot assay. Our findings revealed that CHX caused a rapid disappearance of the mutant BR receptor in *bri1-5* lines, but had a much weaker effect on the BRI1-5 abundance in *sbi3 bri1-5* lines. Similar to observations in *sbi3 bri1-235* and *sbi3 bri1-9* mutants, BRI1 became non-detectable after 9 h of CHX treatment in *bri1-235* and *bri1-9*, but BRI1 abundance level in *sbi3 bri1-235* and *sbi3 bri1-9* was stable (Figures 4D,E and Supplementary Figure 4G). We thus concluded that the observed increased BRI1 abundance in the *sbi3 bri1* mutant is largely caused by attenuated degradation rather than by increased protein biosynthesis, supporting a functional role of SBI3 in the ERAD of the mutant BR receptor.

Endoglycosidase H (Endo H) is capable of cleaving N-glycan of ER-retained proteins but not Golgi-processed complex-type N-glycan. Endo H sensitivity assay using an anti-BRI1 antibody provided an accessible biochemical way to examine the subcellular distribution of BRI1, As shown in Figures 4F,G, a small amount of BRI1-5 and BRI1-235 carrying complex-type N-glycan in *sbi3 bri1-5* and *sbi3 bri1-235*, respectively. This effect was more obvious when the sample volume increased or the exposure time was extended, whereas we failed to detect the complex-type N-glycan in *bri1-5* or *bri1-235* (Figures 4F,G). Consistent with these, BRI1-9 was sensitive, and a minor fraction that was insensitive to Endo H in *sbi3 bri1-9* (Supplementary Figure 4H), likely due to the escape of BRI1-5, BRI1-235, and BRI1-9 from the ER, suggesting that *sbi3* reduces the stringency of quality control of BRI1-5, BRI1-235, and BRI1-9. We also examined eBL-induced changes in the phosphorylation status of BES1, a marker of BR signaling, BES1 was rapidly dephosphorylated in *sbi3 bri1-9*, resembling *sbi3* and Ws-2. However, little change was found in *bri1-5* mutants (Figure 4H).

**FIGURE 5:**
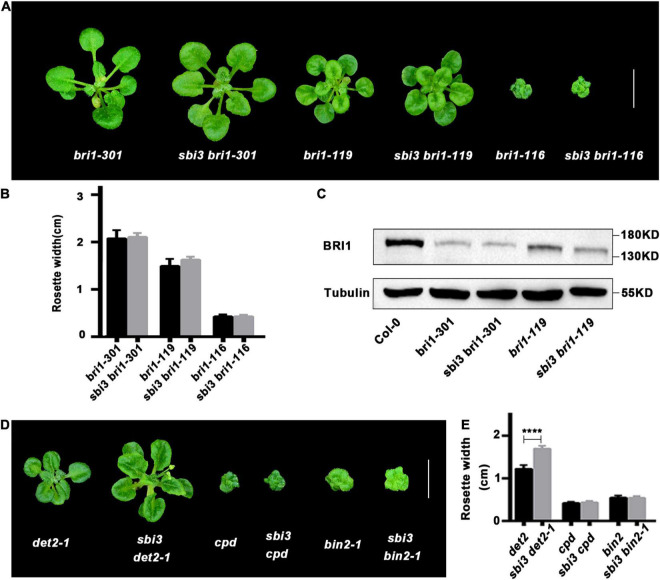
The double mutants of other backgrounds. **(A)** Phenotypes of *bri1-301*, *bri1-119*, *bri1-116* and their corresponding double mutants with *sbi3* grown in soil for 3 weeks. Scale bar, 1 cm. **(B)** Comparison of rosette width of 3-week-old plants measured using the ImageJ software. Error bar represents ± standard deviation (SD), *n* ≥ 10. ***P < 0.001 as two-way ANOVA with Tukey’s multiple comparisons test. **(C)** Western blotting analysis of BRI1 protein abundance in Col-0, *bri1-301*, *sbi3 bri1-301*, *bri1-119*, and *sbi3 bri1-119*. **(D)** Phenotypic comparison of *det2-1, cpd*, *bin2-1* and their corresponding double mutants with *sbi3* grown in soil for 3 weeks. Scale bar, 1 cm. **(E)** The width of rosette leaves of 3-week-old plants were measured using the ImageJ software. Error bar represents ± standard deviation (SD), *n* ≥ 10. ****P < 0.0001 as one-way ANOVA with Tukey’s multiple comparisons test.

To investigate whether *sbi3* also restored other *bri1* mutants, we crossed *sbi3* into several other *bri1* alleles including *bri1–301*, *bri1–119*, and *bri1–116*. The *bri1–301* possesses a missense mutation in the kinase domain of BRI1 (Xu et al., 2008), whereas *bri1–119* mutant contains a mutation in the ID-LRR22 domain (Noguchi et al., 1999) while *bri1-116* is a null allele (Li and Chory, 1997; Friedrichsen et al., 2000). None of these *bri*1 mutants tested was suppressed by *sbi3* (Figures 5A–C). Our results suggest that *sbi3* regulates the abundance of kinase-active and misfolded ER-retained BRI1. Meanwhile, we studied the genetic interaction between *sbi3* and BR-deficient mutants *det2-1* (de-etiolated 2, a weak BR biosynthetic mutant) (Li et al., 1996) and *cpd* (a strong BR biosynthetic mutant) (Szekeres et al., 1996). *sbi3* slightly rescued the growth retardation phenotype of *det2-1*, but it had no effect on *cpd*, probably attributable to a small amount of endogenous BRs in plants (Figures 5D,E). In addition, *sbi3* failed to rescue the growth retardation phenotypes of *bin2-1* (Li and Nam, 2002), indicating that *SBI3* did not act in the BR signaling downstream of *BIN2*.

### The ER Stress Induced by *sbi3*

Previous studies have shown that defective mutations of ERAD often cause the accumulation of aberrant proteins, resulting in activation of the unfolded protein response (UPR) pathway, a highly conserved ER stress response pathway. In this pathway, ER chaperones and ERAD components are upregulated in response to agents tunicamycin (tunicamycin, TM, an ER stress-inducing agent that inhibits protein glycosylation) and dithiothreitol (dithiothreitol, DTT, another widely ER stress-inducer that reduces protein disulfide bonds) to maintain proteostasis, such as BIPs, protein disulfide isomerases (PDIs), calreticulins/calnexin (CRT/CNX) (Su et al., 2011; Huttner et al., 2014b; Lin et al., 2019), OS9/EBS6 (Huttner et al., 2012; Su et al., 2012; Lin et al., 2019), HRD3/SEL1L//EBS5, HRD1 (Su et al., 2011; Lin et al., 2019), and EBS7 (Liu et al., 2015). Furthermore, in mutants lacking ERAD components, such as *hrd3* (*sel1l*), *os9*, *mns4 mns5*, and *pawh1 pawh2* (Liu et al., 2011; Huttner et al., 2012, 2014b; Lin et al., 2019), salt sensitivity was increased.

**FIGURE 6:**
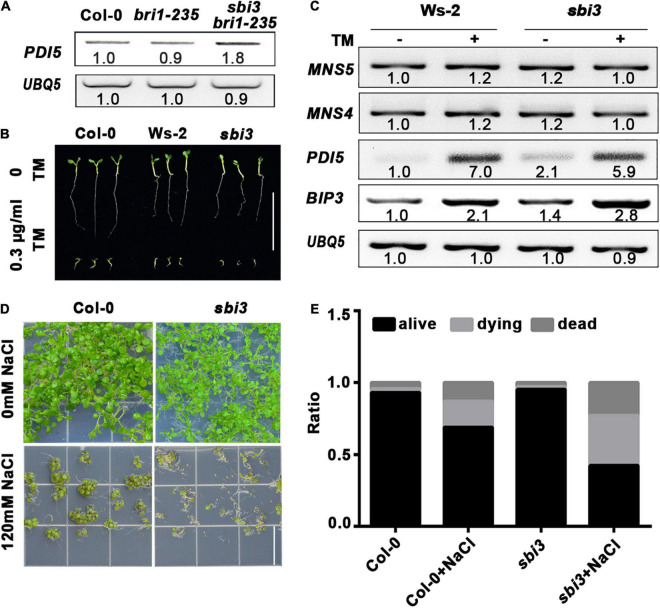
*sbi3* exists UPR and is less tolerant to TM and salt. **(A)** Reverse transcription PCR (RT-PCR) analysis of *PDI5* in Col-0, *bri1-235*, and *sbi3 bri1-235*, *UBQ5* served as a control. **(B)** Comparison of 7-day-old seedlings of wild type and *sbi3* mutant grown in ½ MS with or without 0.3 μg/mL TM. Scale bar = 1 cm. **(C)** Expression levels of *MNS5*, *MNS4*, *PDI5*, and *BIP3* in Ws-2 and *sbi3* with or without 5 μg/mL TM for 6 h. *UBQ5* served as a control. **(D)** The photograph of 12-day-old seedlings grown in ½ MS with or without 120 mM NaCl. Scale bar = 1.5 cm. **(E)** The ratio of the seedlings was shown in the bar graphs, alive (black), dying (light gray), dead (dark gray). These experiments were repeated three times.

We found that the expression abundance of *PDI5* was increased in *sbi3 bri1-235*, indicating that the *sbi3* mutation activates the UPR pathway (Figure 6A). To test whether the *sbi3* mutation affects the plant ER stress tolerance, the seedlings of the Arabidopsis wild type and *sbi3* were grown on ½ MS medium containing 0.3 μg/mL TM, and we found that *sbi3* is less tolerant to TM (Figure 6B). Consistently, RT-PCR analysis showed that the expression of *BIP3* and *PDI5* was clearly higher in *sbi3* seedlings treated with 5 μg/mL TM for 6 h compared to their controls. However, we could not detect expression differences in *BIP3* and *PDI5* in their responses to TM treatment in between Ws-2 and *sbi3* (Figure 6C), in agreement with the response of *mns4-1 mns5-1* to TM treatment (Huttner et al., 2014b). As expected, the expression levels of *MNS4* and *MNS5* were not upregulated with or without TM treatment in Ws-2 and *sbi3* (Figure 6C). In addition, we also found that *sbi3* is less tolerant to salt (Figures 6D,E).

### Different Expression Patterns of *MNS4* and *MNS5* in Arabidopsis

**FIGURE 7:**
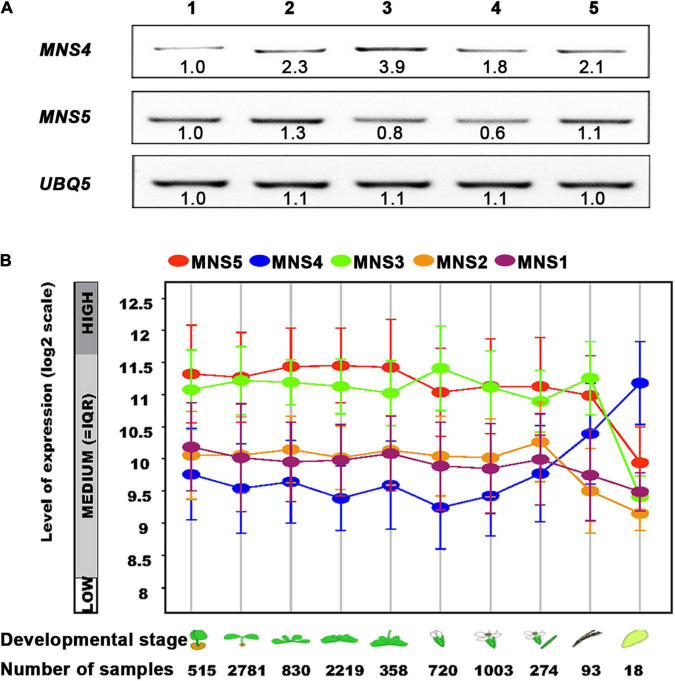
The expression levels of *MNS5* and *MNS4* in different developmental stages. **(A)** The transcripts of *MNS4* and *MNS5* were performed using RT-PCR analysis in WT Ws-2. UBQ5 was a control. 1: 2-weeks-old seedlings, 2: 50-day-old rosette leaves, 3: 50-day-old flowers, 4: 50-day-old siliques, 5: 68-day-old rosette leaves. **(B)** The transcripts comparison of *MNS1*-*MNS5* was plotted as a line graph during the 10 developmental stages from dataset AT_AFFY_ATH1-0 (https://www.genevestigator.com/gv/plant.jsp). The 10 developmental stages: Germinated, young seedling, rosette, developed rosette, bolting and young, flower, developed flower, flowers, and siliques, mature siliques, senescence.

The previous study has revealed that MNS4 is a membrane-bound form while MNS5 is a soluble protein. Furthermore, MNS4 and MNS5 differentially demannosylated the glycoprotein reporters (Huttner et al., 2014b). Here, we showed transcriptional divergence in *MNS4* and *MNS5* across different developmental stages (Figure 7). *MNS4* and *MNS5* transcripts were widely expressed in different developmental stages, and the *MNS5* expression level was higher in the WT seedlings and rosette leaves (Figure 7A). The expression patterns of *MNS1* and *MNS2* transcripts were similar in ten Arabidopsis developmental stages from dataset AT_AFFY_ATH1-0. Conversely, in most cases, the expression level of *MNS5* was higher than that of *MNS4* (Figure 7B). Moreover, we performed the phylogenetic analysis and sequence alignments of the Arabidopsis GH47 family that has three branches, MNS1/MNS2, MNS3 and MNS4/MNS5 (Supplementary Figures 7, 8). The homology analysis revealed that the overall sequence identity between MNS1 and MNS2 was 83.54%, while the identity of MNS4 and MNS5 was 44.10% (Supplementary Figure 7B), suggesting that MNS1 and MNS2 are recently divergent while MNS4 and MNS5 are anciently divergent. In fact, both MNS4 and MNS5 presented in the whole kingdom of plants while both MNS1 and MNS2 presented no earlier than vascular plants (Supplementary Table 2). Taken together, MNS4 and MNS5 likely have a significant functional divergence and our finding is consistent with this assessment, which is a non-redundant function in MNS5 in the regulation of ERAD and ER-stress response.

## Discussion

Endoplasmic Reticulum-Associated Degradation is one of the major processes in maintaining proteostasis. Using misfolded transmembrane receptor kinases, several regulatory components of ERAD have been identified. Previous studies have found that BRI1-9 (an ER-retained Ser662Phe mutation in the ligand-binding domain of BRI1) is ubiquitinated and degraded *via* a classic glycan-dependent, 26S proteasome, and HRD1 complex-mediated pathway after retrotranslocation from the ER into the cytosol (Hong et al., 2009; Liu et al., 2015). On the other hand, the ER-trapped BRI1-5 mutant that carries a Cys69Tyr mutation in the extracellular domain, is regulated by a monoglucosylation (Glc_1_Man_7_GlcNA_2_)-dependent, proteasome-independent ERAD process, but no ubiquitination has been reported so far (Hong et al., 2008; Huttner et al., 2014b). Similar to BRI1-5, SUBEX-C57Y (a misfolded variant of the receptor-like kinase STRUBBELIG’s extracellular domain) is a novel glycoprotein ERAD substrate disposed of by glycan-dependent and non-proteasome dependent route (Huttner et al., 2014a). Unlike the yeast ERAD substrate carboxypeptidase Y, CPY (Jakob et al., 1998; Kostova and Wolf, 2005; Spear and Ng, 2005), none of the three N-glycans on SUBEX-C57Y displays a specific glycan signal for degradation (Huttner et al., 2014a). Furthermore, topologically different folding-defective ERAD substrates do not interfere with the glycan-dependent HRD1 machinery in plants (Shin et al., 2018). Here, we add that the mutant *bri1-235* that harbors a Ser156-to-Phe mutation in the less conserved fourth LRR of BRI1 in Arabidopsis is degraded in a proteasome-independent ERAD way (Figures 2G,H). The mutation of the 156th amino acid in *bri1-235* resulted in the absence of an N-glycosylation site in position 154 (Supplementary Figure 9), which may affect protein folding and degradation (Hou et al., 2019). It would be of great interest to see if ubiquitination is required in the ERAD substrate BRI1-235. Although a new model of the Arabidopsis Hrd1 complex has been depicted by Lin et al. (2019), it’s not clear whether all ERAD substrates are dependent on the Hrd1 complex -containing ERAD machinery in plants.

The N-glycan analysis of the different *mns* mutants confirms that two functionally redundant Golgi-α-mannosidases MNS1 and MNS2 act downstream of MNS3 in the Arabidopsis regular N-glycan processing pathway, which readily cleaves off three-terminal α1,2-linked Man residues from the A- and C-branches of Man8GlcNAc2 substrate, resulting in the formation of Man5GlcNAc2 (Liebminger et al., 2009). The ER-type α-mannosidase MNS3 is required for the efficient biosynthetic role by trimming a terminal α1,2-mannose residue from the middle branch (B-branch) of the Man9GlcNAc2 oligosaccharide in the misfolded proteins, forming a Man8GlcNAc2 isomer (Liebminger et al., 2009). MNS3 contains an amino acid tetrapeptide signal motif (LPYS: leucine, proline, tyrosine, serine) in the cytoplasmic tail, acting as a specific Golgi-localization signal (Schoberer et al., 2019).

The other two Arabidopsis class I α-mannosidases MNS4 and MNS5 are not part of the regular N-glycan processing pathway of properly folded secretory glycoproteins. In the ER, MNS4 and MNS5 with a largely redundant function accelerate the demannosylation of the C-branch to generate a terminal α1,6-linked Man, that acts as the glycan signal for ERAD of misfolded variants of BRI1. MNS4 has a transmembrane segment, while MNS5 is soluble (Huttner et al., 2014b). Consistently, they differentially demannosylate glycoprotein substrates, meaning that they share some but not the other substrates (Huttner et al., 2014b). The expression patterns of *MNS4* and *MNS5* were divergent across 10 Arabidopsis developmental stages from dataset AT_AFFY_ATH1-0. Significantly, in most cases, the expression level of *MNS5* was higher than the corresponding *MNS4* (Figure 7B). Furthermore, MNS4 and MNS5 presented across the whole plant kingdom, meaning that they have been duplicated and diverged since single-cell plants (Supplementary Table 2). This could mean that MNS4 and MNS5 have a distinct function (significantly functional divergence). Yet, a previous study suggested that MNS4 and MNS5 have a redundant function (Huttner et al., 2014b). Our discovery of a non-redundant function in MNS5 offers new insight into the distinct functions of MNS4 and MNS5. Still, forward genetic mutants of MNS4 are highly sought and screening for additional ERAD substrates that can distinguish the functions of MNS4 and MNS5 are also urgently required. In addition, it is worth examining whether sbi3 protein still has the enzyme activity of MNS5 or has an additional activity.

It is worth noting that *mns4-1* or *mns5-1* obtained by T-DNA insertion could not suppress the dwarf of *bri1-5* and *bri1-9*, only the deficiency of both *MNS4* and *MNS5* could (Huttner et al., 2014b). However, our new mutant *sbi3* could directly inhibit the phenotype of *bri1-5*, *bri1-9*, and *bri1-235*. In zebrafish, genetic compensation by the transcriptional upregulation of genes related to a mutated gene had recently been proposed as the possible cause for the observed phenotypical discrepancies in different mutants of the same gene (Rossi et al., 2015). Moreover, El-Brolosy et al. (2019), Ma et al. (2019) and Wilkinson (2019) reported an underlying molecular mechanism of the genetic compensation response, which was specifically triggered by PTC (premature termination codons)-bearing mRNA in mutations.

The RT-PCR analysis had revealed that the *MNS4* transcripts were upregulated in *mns4-1*. However, there was no transcript of *MNS5* in *mns5-1* (Huttner et al., 2014b). We show that transcripts of *MNS4* and *MNS5* were not upregulated in mutant *sbi3*. Yet, *sbi3* has a clear phenotype but *mns5-1* has no detectable phenotype (Figures 1, 2 and Supplementary Figure 4; Huttner et al., 2014b). We thus deduce that the phenotypical discrepancy of *sbi3* and *mns5-1* are attributed to genetic compensation in *mns5-1*. Yet, the genetic compensation in *mns5-1* might not be caused by the transcription upregulation of the related gene, namely no upregulation of the transcripts of *MNS5* (*mns5-1*) and *MNS4* in *mns5-1*. Therefore, the exact cause of the phenotypical discrepancy of *sbi3* and *mns5-1* is currently unknown, which is certainly a major part of our future endurance. Nevertheless, our finding raises awareness that the traditional forward genetic approach might still be necessary to complement the T-DNA or CRISPR-Cas9 systems on the study of gene functions *in planta*.

## Data Availability Statement

All datasets generated for these findings are available in the main text and the Supplementary Material, further inquiries can be directed to the corresponding author.

## Author Contributions

GW and XS conceived, designed, and coordinated the project. XS and CG performed the genetic mapping and the whole genome re-sequencing. XS and QZ performed protein expression and purification. QW and YZ performed the bioinformatics analysis. GW, XS, and KA wrote the original draft and other authors read and edited the manuscript. All authors interpreted the results.

## Conflict of Interest

The authors declare that the research was conducted in the absence of any commercial or financial relationships that could be construed as a potential conflict of interest.

## Publisher’s Note

All claims expressed in this article are solely those of the authors and do not necessarily represent those of their affiliated organizations, or those of the publisher, the editors and the reviewers. Any product that may be evaluated in this article, or claim that may be made by its manufacturer, is not guaranteed or endorsed by the publisher.

## Footnotes

1
http://rsb.info.nih.gov/ij
