# Supplementary material for: A Non-redundant Function of MNS5: A Class I α-1, 2 Mannosidase, in the Regulation of Endoplasmic Reticulum-Associated Degradation of Misfolded Glycoproteins
Source: Front Plant Sci. 2022 Apr 19;13:873688. doi: 10.3389/fpls.2022.873688 (PMC9062699; doi:10.3389/fpls.2022.873688)
Supplement: Supplementary file 1 [file Data_Sheet_1.pdf]

Supplementary figures and tables:

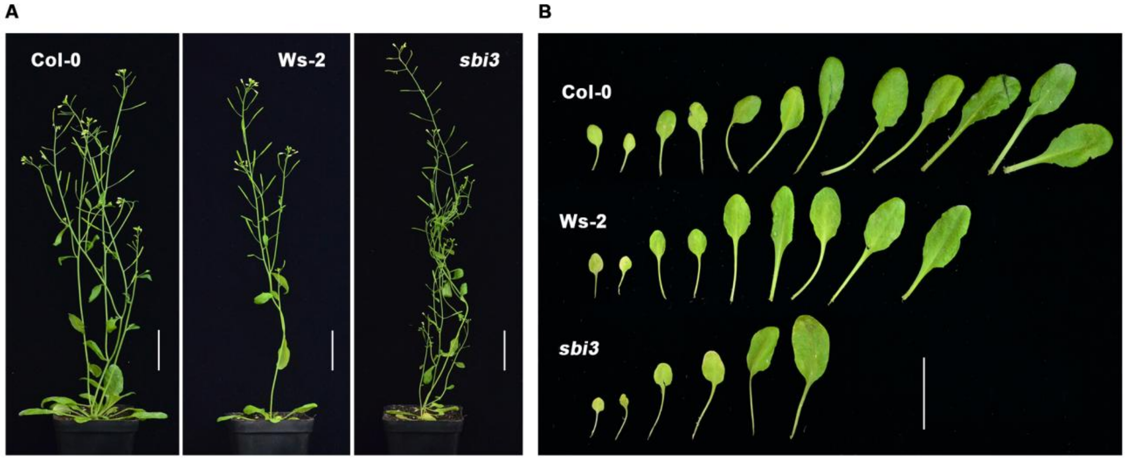

Supplementary Figure 1. Phenotypes of *Arabidopsis* wild type and the mutant *sbi3*. (A) Growth comparison of 2-month-old mature plants of Ws-2, Col-0 and the mutant *sbi3*. Scale bar = 3 cm. (B) Fewer leaves were shown in *sbi3* than wild type. Scale bar = 2 cm.

	Ws-2	*bri1-5*	*sbi3 bri1-5*	*sbi3*	Col-0	*bri1-9*	*sbi3 bri1-9*	*bri1-235*	*sbi3 bri1-235*
Lobe (μm)	17.4±0.48	13.4±0.29 ****	15.3±0.36 **	18.92±0.51	19.41±0.26	15.22±0.31 ****	17.99±0.29 ****	13.96±0.37 ****	15.79±0.38 *
Neck (μm)	17.98±0.26	18.81±0.31	18.49±0.39	17.72±0.37	17.41±0.18	19.66±0.31 ****	18.94±0.33 **	18.73±0.44	17.94±0.29
Perimeter (μm)	454.6±17.7	301.4±10.2 ****	400.8±13.7 ***	505.6±22.2	460.4±13.2	304.2±9.57 ****	403.3±11.2 ***	386±7.71 **	414.4±13.9
Area ($μm^2$)	4831±187	3146±126 ****	3876±146 *	4983±257	4875±105	2763±141 ****	4207±146 ****	3987±127 ***	4171±116
Circularity	0.31±0.01	0.45±0.02 ****	0.31±0.01 ****	0.26±0.01	0.32±0.01	0.37±0.01 *	0.33±0.01 *	0.34±0.01	0.32±0.02

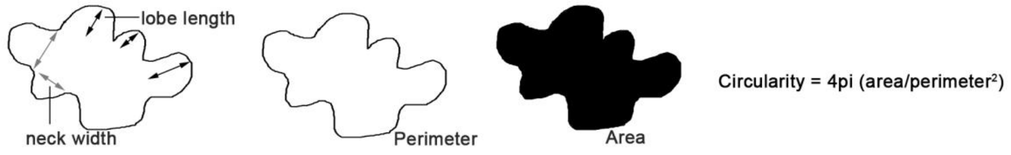

Supplementary Figure 2. The size of cotyledon pavement cells.

The size measurements of cotyledon pavement cells were performed using ImageJ software. N⩾30 for measurements. Tukey's multiple comparisons test: *P<0.05, **P<0.01, ***P<0.001, ****P<0.0001.

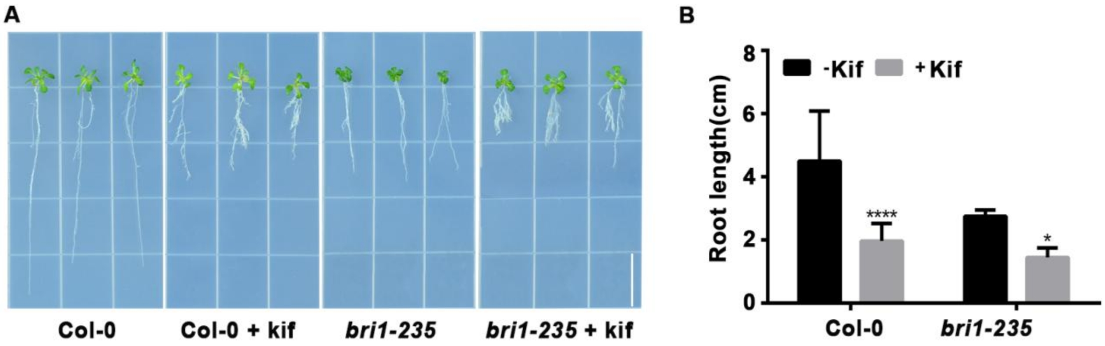

Supplementary Figure 3 The sensitivity of *bri1-235* to Kif was detected. (A) One-week-old seedlings were transferred to half-strength MS medium supplemented with or without 10 μM Kif for continued growth. Phenotypes were photographed and analyzed 9 days later. Scale bar = 1.5 cm. (B) The quantitative analysis of root length of seedlings shown in (A). N = 10 seedlings. *P < 0.05 and ****P < 0.0001 as two-way ANOVA with Sidak's multiple comparisons test.

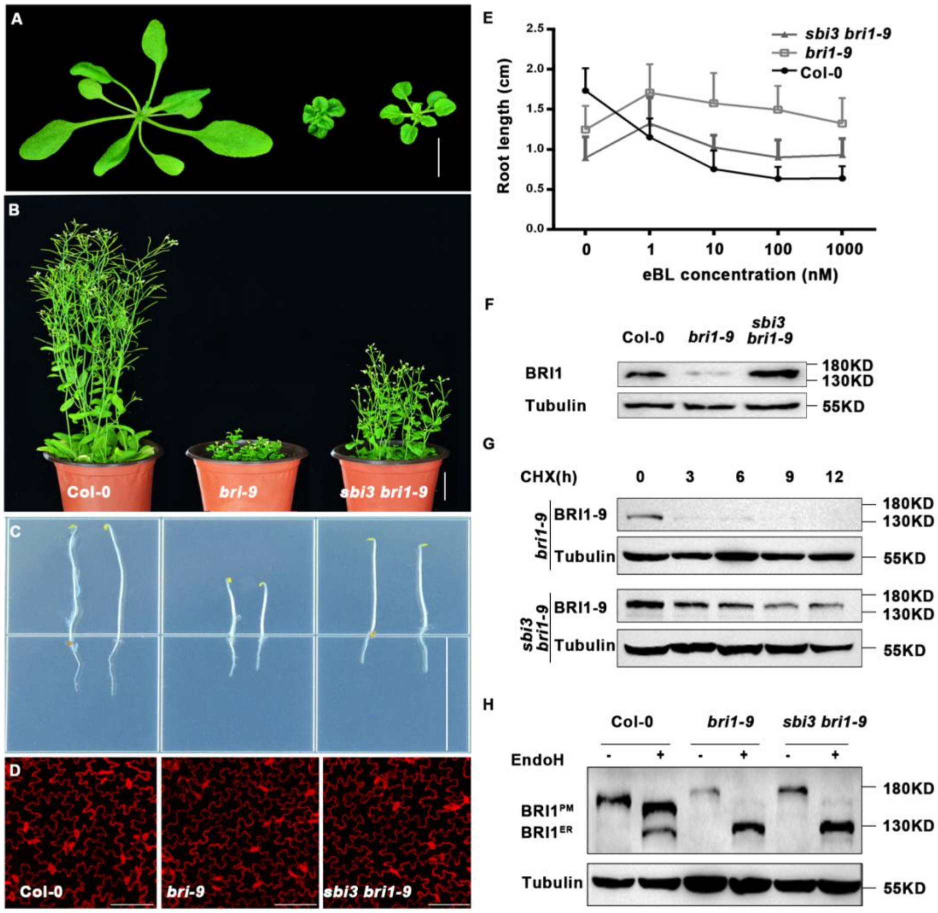

Supplementary Figure 4. The *sbi3* mutation inhibits the ERAD of *bri1-9*. (A) Phenotypes of three-week-old soil-grown seedlings of Col-0, *bri1-9* and *sbi3 bri1-9*. Scale bar, 1 cm. (B) Phenotypes of 2-month-old mature plants of Col-0, *bri1-9* and *sbi3 bri1-9*. Scale bar, 3 cm. (C) Hypocotyl comparison of 5-day-old dark-grown seedlings of Col-0, *bri1-9* and *sbi3 bri1-9*. Scale bar, 1.5 cm. (D) The morphology of cotyledon pavement cells from Col-0, *bri1-9* and *sbi3 bri1-9*. Scale bar, 100 µm. (E) The 24-eBL-induced root inhibition assay. Quantitative analysis of root length was plotted as line graph, n ≥ 30 seedlings. Error bar denotes ± standard deviation (SD), three independent assays. (F) Western blot analysis of BRI1 protein abundance in Col-0, *bri1-9* and *sbi3 bri1-9*. (G) Immunoblot analysis of bri1-9 stability in *bri1-9* and *sbi3 bri1-9* with the anti-BRI1 antibody. Two-week-old seedlings were treated with 180 µM CHX for indicated incubation times. (H) EndoH analysis of Col-0, *bri1-9* and *sbi3 bri1-9*. $BRI1^{ER}$ is the ER-localized proteins form, while $BRI1^{PM}$ denotes the localization of BRI1 proteins in the plasma membrane.

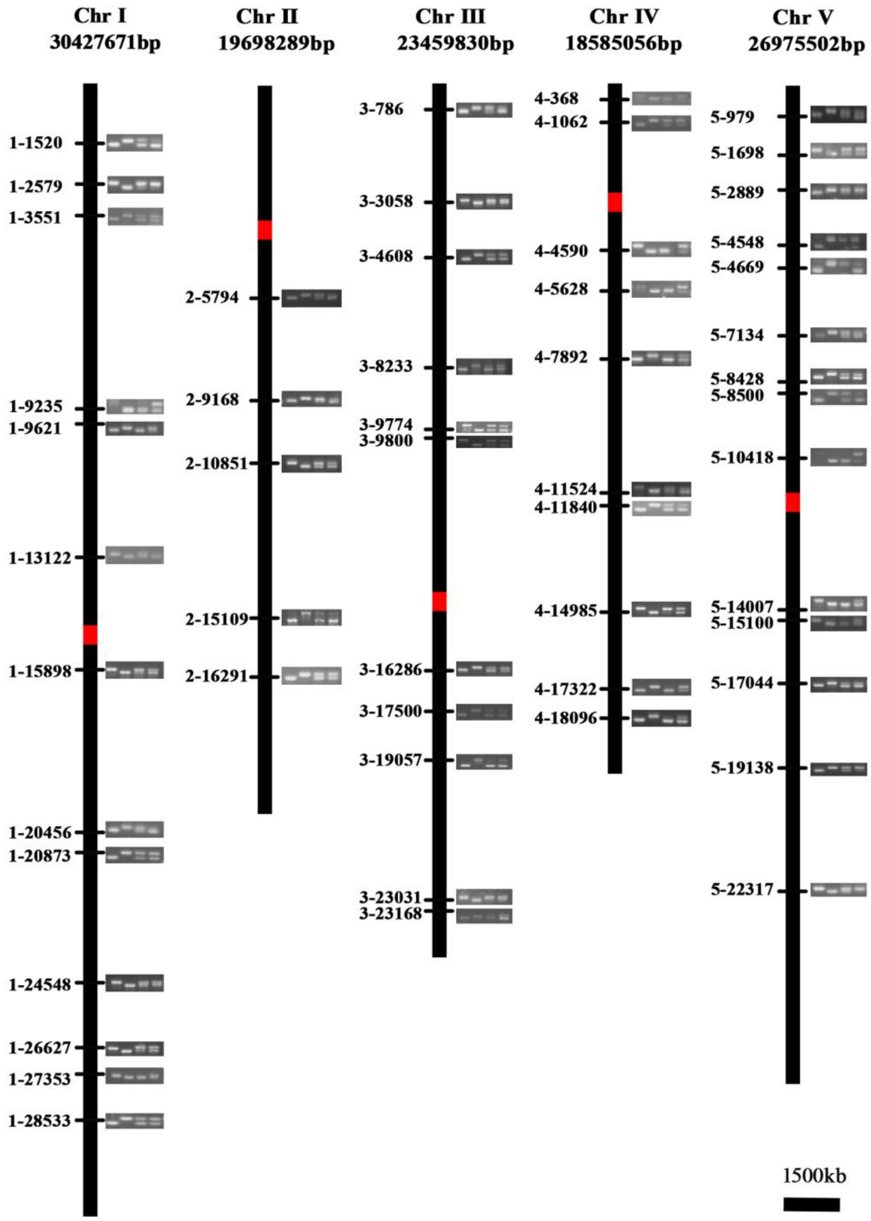

Supplementary Figure 5. The preliminary mapping of the *sbi3* mutation site. The experimental results of bulked segregant analysis (BSA) showed that the mutant gene was located on the first chromosome. DNA samples from left to right are Ws-2, Col-0, Bulk, Ws-2+Col-0.

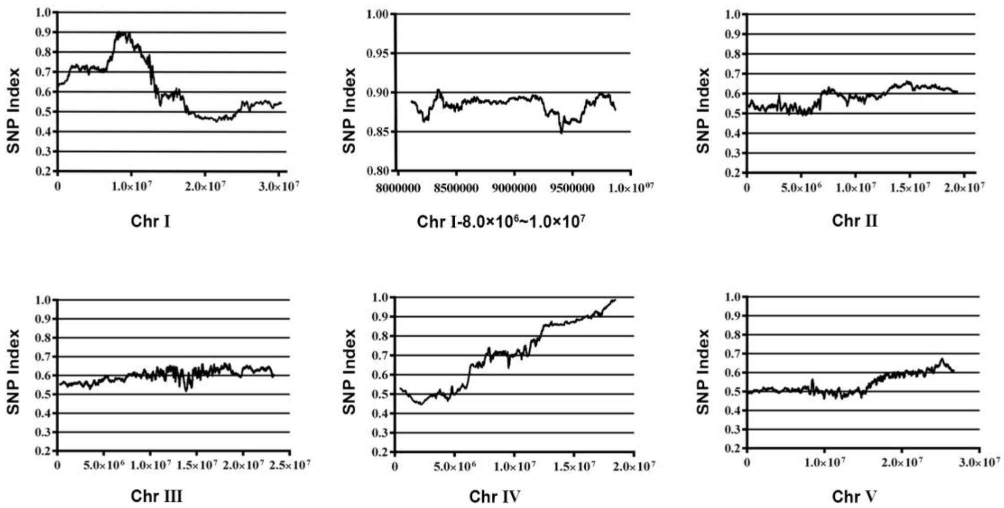

Supplementary Figure 6. SNP-index plots for all *Arabidopsis thaliana* 5 chromosomes using MutMap method. SNP-index = ALT depth/ (REF depth + ALT depth). ALT (altered base) depth and REF (reference base) depth were given by Beijing Nuohe Zhiyuan Technology Co., Ltd. The x-axis represents the position of the chromosomes.

A

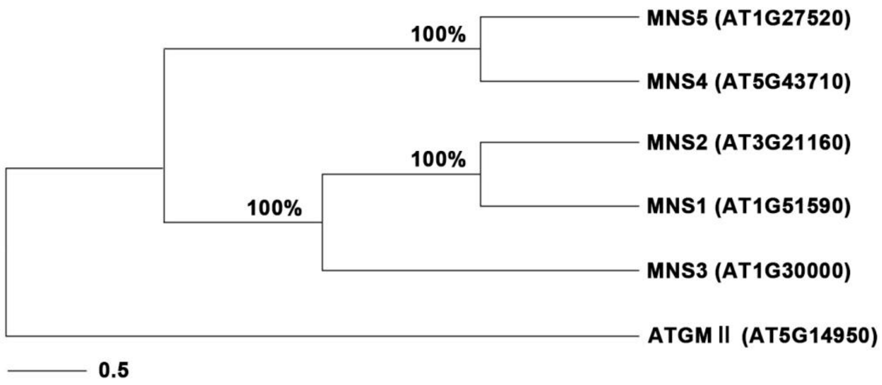

B

Full-length	MNS5	MNS4	MNS3	MNS2	MNS1
**MNS5**		**44.10**	30.32	31.03	33.77
**MNS4**	**44.10**		29.00	30.40	30.90
**MNS3**	30.32	29.00		43.10	42.24
**MNS2**	31.03	30.40	43.10		83.54
**MNS1**	33.77	30.90	42.24	83.54	

Supplementary Figure 7. The comparison of Arabidopsis GH47 family: MNS1-MNS5. (A) The phylogenetic analysis of the Arabidopsis GH47 family: MNS1-MNS5. ATGMⅡ was one of GH38 family. The protein sequences were aligned with MEGA 5.2 software, and the phylogenetic tree was generated using the Neighbor-Joining method. Bootstrap values: 1000 replicates. (B) The homology comparison of AtMNS1-MNS5 amino acids was constructed with ClustalW (http://www.clustal.org/).

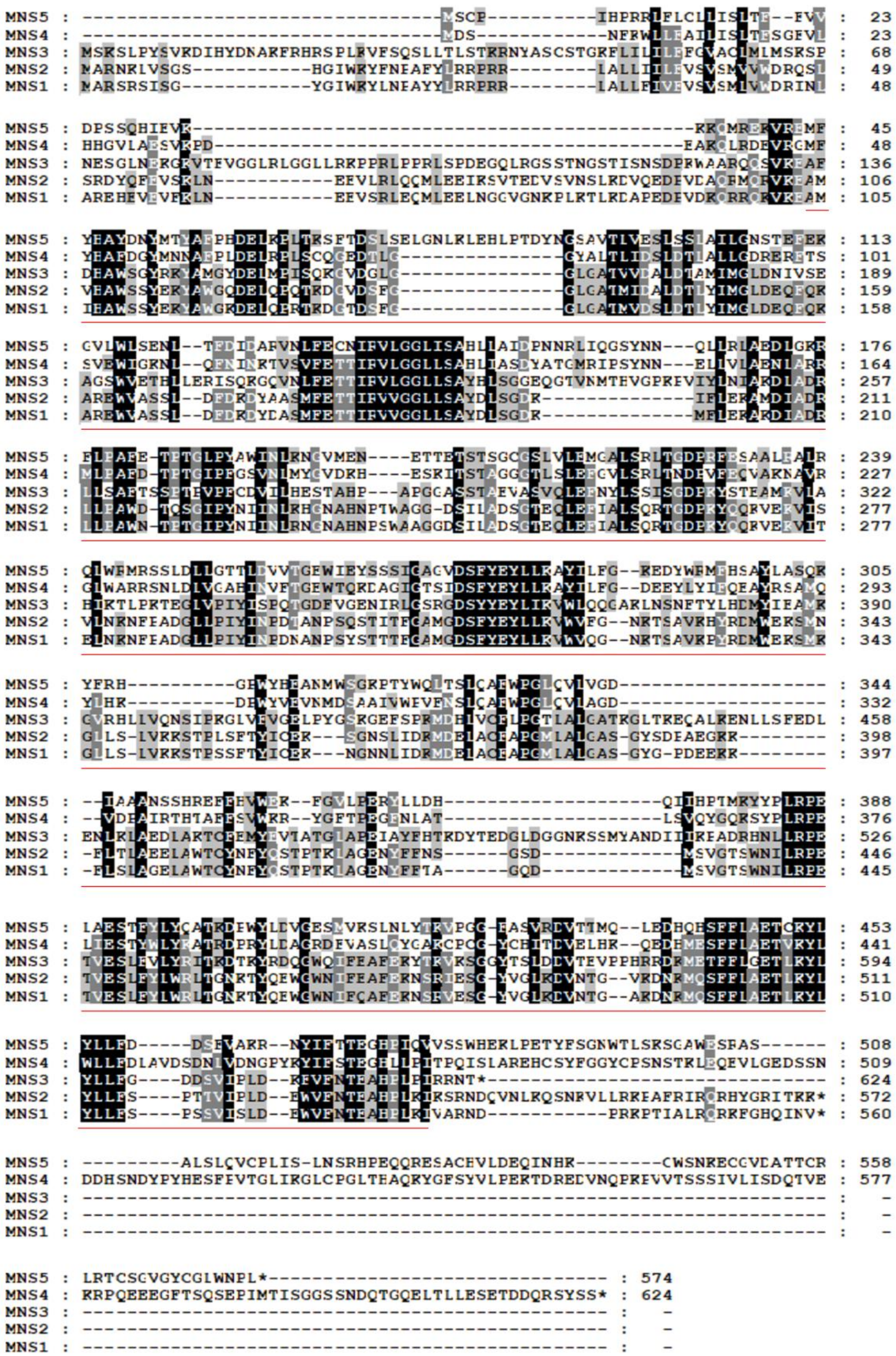

Supplementary Figure 8. Structure-based sequence alignments of AtMNS1-5. GH47 domains were presented in red line.

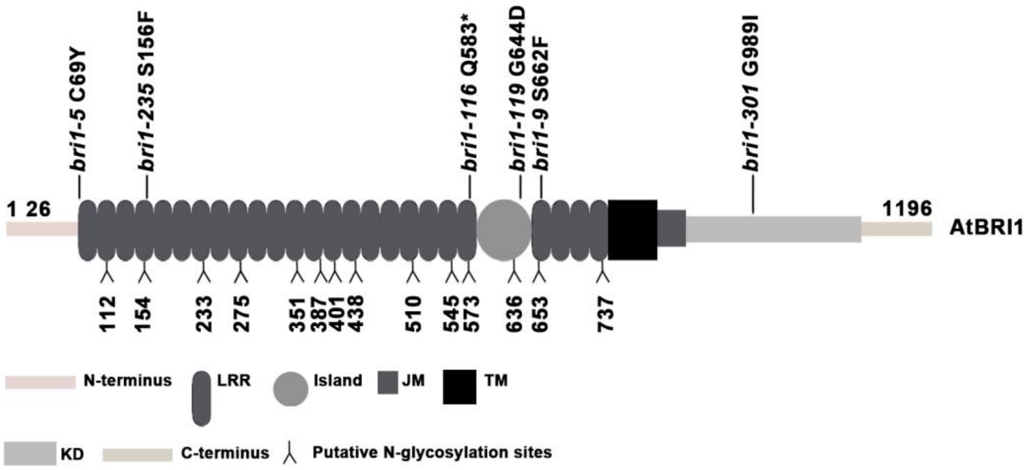

Supplementary Figure 9. The schematic structure of BR receptor BRI1. All *bri1* mutants used in this study were listed and labeled. *bri1-5*, *bri1-9*, *bri1-119* (Noguchi et al., 1999), *bri1-235* (Hou et al., 2019), *bri1-301* (Xu et al., 2008), *bri1-116* (Friedrichsen et al., 2000). The putative N-glycosylation sites in the N-terminal extracellular domain of BRI1 also were listed (http://www.cbs.dtu.dk/services/NetNGlyc/#opennewwindow).

Supplementary Table 1: Primer pairs used in this study.

Name	Sequence (5´-3´)
1-1520F	CATGGGCTTTAGGCCATTTA
1-1520R	TGAGACTTACGAGGATGAAACAA
1-2579F	CTCTTGGTGGTGTCCCAAGT
1-2579R	TCGACGCAGTTTTTCATCAG
1-3551F	ACCCAAGTGATCGCCACC
1-3551R	AACCAAGGCACAGAAGCG
1-9235F	CCCTTCCATTGAACAGAATGA
1-9235R	TAAATCTGTGTCCCGCCAAA
1-9621F	AGGTTTTATTGCTTTTCACA
1-9621R	CTTTCAAAAGCACATCACA
1-13122F	ATCAAAGGTTGCCACAAATG
1-13122R	TTCGGTTTGATTTGGGTTTC
1-15898F	ATGACAGCATTCCAACAAACT
1-15898R	TGAAAGGAAAAATCAGAATTCCA
1-20456F	GGCTCCATAAAAAGTGCACC
1-20456R	CTGATCTCACGGACAATAGTGC
1-20873F	GGCTCCATAAAAAGTGCACC
1-20873R	CTGATCTCACGGACAATAGTGC
1-24548F	ACAAAATGCCGATCCAACAT
1-24548R	TGCTGAAAACGTCAAGACCA
1-26627F	GCAATTCATCAGCAGGAGGT
1-26627R	ATCAGGGAGCAAAATGCAAG
1-27353F	TGTTTTTTAGGACAAATGGCG
1-27353R	CTCCAGTTGGAAGCTAAAGGG
1-28533F	GTTCACAGAGAGACTCATAAACCA
1-28533R	CTGGGAACGGTTCGATTCGAGC

2-5794F	TCATGCGGAAGTGAGTGTTC
2-5794R	TGCTTGAGTTTGGTTTTTGC
2-9168F	CGGAAACGAAGACGAGTGAT
2-9168R	CGCCCCCTAATTTTTCTTTT
2-10851F	CAATTGGGTACGCAAGATCA
2-10851R	CACTCACTTTTTGCGTTTGC
2-15109F	GCCGTCAATGTTTGCTTTTA
2-15109R	AATAAGCTGAGGAGGCAGCA
2-16291F	GAGGACATGTATAGGAGCCTCG
2-16291R	TCGTCTACTGCACTGCCG
3-786F	CATCCGAATGCCATTGTTC
3-786R	AGCTGCTTCCTTATAGCGTCC
3-3058F	GGATGCGAAATAAGCGATGA
3-3058R	GGTGTAGCCGGCGTAAGTAA
3-4608F	CTCTGTCACTCTTTTCCTCTGG
3-4608R	CATGCAATTTGCATCTGAGG
3-8233F	TCACAATTCGATCGTCATTACA
3-8233R	GGAATGTCATGTATGTATCTCTTGG
3-9774F	CCCCGAGTTGAGGTATT
3-9774R	GAAGAAATTCCTAAAGCATTC
3-9800F	CCCCGAGTTGAGGTATT
3-9800R	GAAGAAATTCCTAAAGCATTC
3-16286F	GGTTTGGTGGGAGAGAATGA
3-16286R	CAAAAGAAATGCAACGAGACA
3-17500F	TTTGTCTGAAGATGTGGAGAGAGAG
3-17500R	CAAAACCCCACTCTTCATTATTGTT
3-19057F	GTTCTCTGCATTCCACACATACTCT
3-19057R	CTTGGGTATTCTGAAGAGCATAAAT

3-23031F	ATGGAGAAGCTTACACTGATC
3-23031R	TGGATTTCTTCCTCTCTTCAC
3-23168F	GCAATGCGCAAAAGCAATCAGAACA
3-23168R	GTTGAGAATTCACTGTTTTTACTGA
4-368F	GCCGCTCAACATGCTATAAA
4-368R	TGTGTGGTCAGGAATTAACAAA
4-1062F	TCATGACTATCCTTGTGCAGAT
4-1062R	TGATTTTGGTTTTGATTTTGACC
4-4590F	TGGCTTTCGTTTATAAACATCC
4-4590R	GAGGGCAAATCTTTATTTCGG
4-5628F	TGGCTTTCGTTTATAAACATCC
4-5628R	GAGGGCAAATCTTTATTTCGG
4-7892F	CTCGTAGTGCACTTTCATCA
4-7892R	CACATGGTTAGGGAAACAATA
4-11524F	AATTTGGAGATTAGCTGGAAT
4-11524R	CCATGTTGATGATAAGCACAA
4-11840F	ATTTACGGCGGTTCTTGATG
4-11840R	TGCACCACACACATTCTCCT
4-14985F	TGGCTGCAGCGAATAACTAA
4-14985R	TGGTGCTGGTGAAACCAATA
4-17322F	TCCTAGATTCGTGGGGTTTG
4-17322R	CGCTTAAAGCTGCAAGAACC
4-18096F	CGACGAATCGACAGAATTAGG
4-18096R	GCGAAAAAACAAAAAAATCCA
5-979F	CCACTTGTTTCTCTCTCTAG
5-979R	TATCAACAGAAACGCACCGAG
5-1698F	ACCTGAACCATCCTCCGTC
5-1698R	TCATTTTGGCCGACTTAGC

5-2889F	GGATCCCTAACTGTAAAATCCC
5-2889R	TACCGTCAATTTCATCGCC
5-4548F	CAGTCTAAAAGCGAGAGTATGATG
5-4548R	GTTTTGGGAAGTTTTGCTGG
5-4669F	CAGTCTAAAAGCGAGAGTATGATG
5-4669R	GTTTTGGGAAGTTTTGCTGG
5-7134F	AACGCCGGAGTTAGTCGAT
5-7134R	CGTATATGGATTGCGTGACAA
5-8428F	GGTTTCGTTTCACTATCCAGG
5-8428R	AGAGCTACCAGATCCGATGG
5-8500F	GGTTTCGTTTCACTATCCAGG
5-8500R	AGAGCTACCAGATCCGATGG
5-10418F	AGGCATGGGAGACATTTACG
5-10418R	GGAGAAAATGTCACTCTCCACC
5-14007F	AAACTCGAGAGTTTTGTCTAGATC
5-14007R	CTCAGAGAATTCCCAGAAAAATCT
5-15100F	TGATGTTGATGGAGATGGTCA
5-15100R	CTCCACCAATCATGCAAATG
5-17044F	CAGACGTATCAAATGACAAATG
5-17044R	GACTACTGCTCAAACTATTCGG
5-19138F	AACTCATGCAATGCGACATC
5-19138R	CCCGTCCATGATCTGTTTCT
5-22317F	GCATTGAAATAGTGTTTTTAACCAAA
5-22317R	TGTTGGTTGCCACCTTATCA
Actin2-RT-F	ACTCTCCCGCTATGTATGTCG
Actin2-RT-R	TGGACCTGCCTCATCATACTC
BRI1-RT-F	GGTAGAGAGATGAGGAAGAGA
BRI1-RT-R	CTCATAATTTTCCTTCAGGAACTTC

CPD-RT-F	GTTCTTATCCTGCTTCCATTTG
CPD-RT-R	AGCCACTCGTAGCGTCTCATT
DWF4-RT-F	CGAAGGAAGGCTCTTTGAATG
DWF4-RT-R	CTTCAACGGCTTTAGGGCAA
BAS1-RT-F	GTTCAGGACATTGTGGAGGAG
BAS1-RT-R	GGATAAAGCAACATAAGGACG
SBI3/MNS5-KpnI -F1	CGGTACCATGTCTTGTCCTATCCATCCTAGGC
SBI3/MNS5-BamHI -R1	CGGATCCTAAGGGATTCCATAAGCCGCAGTATC
SBI3/MNS5-clone-F2	CATGGGCCTTGGTATCATGAAGCTAA
SBI3/MNS5-clone-R2	TTAGCTTCATGATACCAAGGCCCATG
AT1G27570-dCAPS-F	TCACAATAGCCTGGTCGTGTAGTAGGGGTGAGA
AT1G27570-dCAPS-R	AGACCGGCAGCAAGGGAGGAAAT
AT1G27520-dCAPS-F	GCCTTGGTAATATACTAGCCTGAA
AT1G27520-dCAPS-R	ATCTTCCAATTGCATGGTCG
UBQ5-RT-F	AACCCTTGAGGTTGAATCATC
UBQ5-RT-R	CTCCTTCTTTCTGGTAAACGT
BiP3-RT-F	TTCGAAGTGGACGCAAATGG
BiP3-RT-R	CTATAGCTAACCGACTCAAAAGACCT
PDI5-RT-F	CTCGTGAAGCTGAGGGTATTG
PDI5-RT-R	TGTGCGAAATCTAACTCAGAG
MNS5-RT-F	TTGGAAAAGAAGACTACTGGCGAATGT
MNS5-RT-R	GCGAGAAAGAAACTGTGCTGATGA
MNS4-RT-F	AGGCTATGCGTTGACTCTGATTGAC
MNS4-RT-R	CGAGATTTGAACGACGTGCCCATAGT
bri1-5-genotyping-F	AGAATCAAGAGATAGGTGGTTGGG
bri1-5-genotyping-R	GCTGGTTAAAGAAGCAGAGCA
bri1-9-genotyping-F	TAACAATGGTTCGATGATGTTTCTGGACAAGC
bri1-9-genotyping-R	GCATAGTAAGAGCTGACATAGCCTG

bri1-235-genotyping-F	TCTCAACGTCGGATTCAGTGC
bri1-235-genotyping-R	AAGATGTTGCAGAGCAGAGC
bri1-301-genotyping-F	CATCGAAATCTTGTGCCTCTTCTTG
bri1-301-genotyping-R	CTCGGGGTCAAACACATCGCTAATC
bri1-119-genotyping-F	ACCCTTGTAATATCACTAGCAGAGTCTATGAAG
bri1-119-genotyping-R	CGATTTCCGTAAGCATAGTAAGAGC
bri1-116-genotyping-F	TGGCGAGTTACCGATGGATACG
bri1-116-genotyping-R	CTCTTAGATCACCTACCTCATCAGG
det2-1-genotyping-F	AGGATGACTACGAAGACGGAAAC
det2-1-genotyping-R	CAGCCCAGCCCAACCACTCAATAAGCT
LBb1.3	ATTTTGCCGATTTCGGAAC
cpd-22-LP	TTTCTTTCTCTCCGCTCCTTC
cpd-22-RP	CTACTCCGCCGTACACGTTAC

Supplementary Table 2. The presence or absence of MNS1-5 in selected species.

<table>
<tr><th></th><th>MNS1</th><th>MNS2</th><th>MNS3</th><th>MNS4</th><th>MNS5</th></tr>
<tr><td>Botryococcus braunii</td><td colspan="2">5</td><td>1</td><td>1</td><td>1</td></tr>
<tr><td>Chlamydomonas reinhardtii</td><td colspan="2">1</td><td>0</td><td>1</td><td>0</td></tr>
<tr><td>Chromochloris zofingiensis</td><td colspan="2">3</td><td>1</td><td>1</td><td>0</td></tr>
<tr><td>Coccomyxa subellipsoidea</td><td colspan="2">3</td><td>1</td><td>1</td><td>1</td></tr>
<tr><td>Dunaliella salina</td><td colspan="2">0</td><td>0</td><td>1</td><td>0</td></tr>
<tr><td>Micromonas pusilla</td><td colspan="2">2</td><td>0</td><td>0</td><td>1</td></tr>
<tr><td>Micromonas sp RCC299</td><td colspan="2">1</td><td>1</td><td>0</td><td>1</td></tr>
<tr><td>Ostreococcus lucimarinus</td><td colspan="2">1</td><td>0</td><td>1</td><td>0</td></tr>
<tr><td>Volvox carteri</td><td colspan="2">1</td><td>0</td><td>0</td><td>0</td></tr>
<tr><td>Marchantia polymorpha</td><td>1</td><td>0</td><td>1</td><td>1</td><td>1</td></tr>
<tr><td>Physcomitrium patens</td><td>2</td><td>0</td><td>1</td><td>1</td><td>0</td></tr>
<tr><td>Sphagnum fallax</td><td>3</td><td>0</td><td>1</td><td>1</td><td>0</td></tr>
<tr><td>Ceratopteris richardii</td><td>1</td><td>0</td><td>1</td><td>1</td><td>1</td></tr>
<tr><td>Selaginella moellendorffii</td><td>1</td><td>1</td><td>1</td><td>1</td><td>2</td></tr>
<tr><td>Picea abies</td><td colspan="2">1 | 5 | 1</td><td>1</td><td>1</td><td>3</td></tr>
<tr><td>Thuja plicata</td><td>2</td><td>0</td><td>1</td><td>1</td><td>1</td></tr>
<tr><td>Ananas comosus</td><td>2</td><td>0</td><td>1</td><td>1</td><td>1</td></tr>
<tr><td>Musa acuminata</td><td>3</td><td>0</td><td>2</td><td>1</td><td>3</td></tr>
<tr><td>Hordeum vulgare</td><td>1</td><td>0</td><td>1</td><td>1</td><td>1</td></tr>
</table>

Oryza sativa	1	0	1	1	1
Triticum aestivum	3	0	4	5	3
Brachypodium stacei	1	0	1	1	1
Brachypodium distachyon	1	0	1	1	1
Panicum virgatum	6	0	2	3	3
Setaria italica	2	0	1	1	1
Setaria viridis	2	0	1	1	1
Sorghum bicolor	1	0	1	1	1
Zea mays	4	0	1	1	1
Panicum hallii	2	0	1	1	1
Aquilegia coerulea	1	0	1	1	1
Amborella trichopoda	1	0	1	1	1
Amaranthus hypochondriacus	2	0	1	1	1
Kalanchoe fedtschenkoi	2	0	1	1	1
Kalanchoe laxiflora	3	0	2	2	2
Daucus carota	2	0	1	1	1
Mimulus guttatus	1	0	1	1	1
Solanum lycopersicum	2	0	1	1	1
Solanum tuberosum	2	0	1	1	1
Eucalyptus grandis	2	0	1	1	1

Vitis vinifera	3	0	1	1	1
Cicer arietinum	2	0	1	1	1
Cucumis sativus	1	0	1	1	1
Fragaria Vesca	2	0	1	2	1
Glycine max	4	0	2	1	2
Malus domestica	3	0	2	1	4
Phaseolus vulgaris	3	0	1	1	3
Prunus persica	2	0	1	1	1
Trifolium pratense	3	0	1	1	1
Carya illinoinensis	2	0	1	1	1
Vigna unguiculat	1	0	1	1	1
Corymbia citriodora	3	0	1	1	1
Linum usitatissimum	5	0	2	2	2
Manihot esculenta	3	0	2	1	1
Populus trichocarpa	2	0	1	1	2
Ricinus communis	2	0	1	1	1
Salix purpurea	3	0	1	1	2
Anacardium occidentale	2	0	1	2	1
Carica papaya	3	0	1	1	1
Theobroma cacao	2	0	1	1	1

Arabidopsis halleri	1	1	1	1	2
Arabidopsis lyrata	1	1	1	1	1
Arabidopsis thaliana	1	1	1	1	1
Boechera stricta	1	1	1	1	1
Capsella grandiflora	1	1	1	1	1
Capsella rubella	1	1	1	1	1
Eutrema salsugineum	1	1	1	1	1
Lunaria annua	2	1	2	1	1
Stanleya pinnata	3	2	2	2	2
Brassica oleracea capitata	1	2	2	1	0
Brassica rapa	2	2	2	1	1
Sinapis alba	2	2	2	1	1
Myagrum perfoliatum	1	1	1	1	1
Gossypium raimondii	2	0	2	1	1
Citrus sinensis	2	0	0	1	1
Citrus clementina	1	0	1	1	1

The presence or absence of MNS1-5 in selected species, as determined by a BLAST search using sequences of AtMNS1-5 (https://phytozome-next.jgi.doe.gov/blast-search; https://congenie.org/start).

Friedrichsen, D.M., Joazeiro, C.A., Li, J., Hunter, T., and Chory, J. (2000). Brassinosteroid-insensitive-1 is a ubiquitously expressed leucine-rich repeat receptor serine/threonine kinase. *Plant Physiol* 123(4)**,** 1247-1256. doi: 10.1104/pp.123.4.1247.

Hou, Q., Saima, S., Ren, H., Ali, K., Bai, C., Wu, G., et al. (2019). Less Conserved LRRs Is Important for BRI1 Folding. *Front Plant Sci* 10**,** 634. doi: 10.3389/fpls.2019.00634.

Noguchi, T., Fujioka, S., Choe, S., Takatsuto, S., Yoshida, S., Yuan, H., et al. (1999). Brassinosteroid-insensitive dwarf mutants of Arabidopsis accumulate brassinosteroids. *Plant Physiol* 121(3)**,** 743-752. doi: 10.1104/pp.121.3.743.

Xu, W., Huang, J., Li, B., Li, J., and Wang, Y. (2008). Is kinase activity essential for biological functions of BRI1? *Cell Res* 18(4)**,** 472-478. doi: 10.1038/cr.2008.36.
